# Nanobiochar and Copper Oxide Nanoparticles Mixture Synergistically Increases Soil Nutrient Availability and Improves Wheat Production

**DOI:** 10.3390/plants12061312

**Published:** 2023-03-14

**Authors:** Muhammad Imtiaz Rashid, Ghulam Abbas Shah, Maqsood Sadiq, Noor ul Amin, Arshid Mahmood Ali, Gabrijel Ondrasek, Khurram Shahzad

**Affiliations:** 1 Center of Excellence in Environmental Studies, King Abdulaziz University, Jeddah 21589, Saudi Arabia; 2 Department of Agronomy, Pir Mehr Ali Shah Arid Agriculture University, Rawalpindi 46300, Pakistan; 3 Department of Environmental Science, Sub-Campus, COMSATS University Islamabad, Vehari 61000, Pakistan; 4 Department of Chemical and Materials Engineering, Faculty of Engineering, King Abdulaziz University, Jeddah 21589, Saudi Arabia; 5 Department of Soil Amelioration, Faculty of Agriculture, University of Zagreb, 10000 Zagreb, Croatia

**Keywords:** crop attributes, microbial activity, nanofertilizers, nutrient utilization efficiency, smart fertilizers, soil quality

## Abstract

Recently, nanomaterials have received considerable attention in the agricultural sector, due to their distinctive characteristics such as small size, high surface area to volume ratio, and charged surface. These properties allow nanomaterials to be utilized as nanofertilizers, that can improve crop nutrient management and reduce environmental nutrient losses. However, after soil application, metallic nanoparticles have been shown to be toxic to soil biota and their associated ecosystem services. The organic nature of nanobiochar (nanoB) may help to overcome this toxicity while maintaining all the beneficial effects of nanomaterials. We aimed to synthesize nanoB from goat manure and utilize it with CuO nanoparticles (nanoCu) to influence soil microbes, nutrient content, and wheat productivity. An X-ray diffractogram (XRD) confirmed nanoB synthesis (crystal size = 20 nm). The XRD spectrum showed a distinct carbon peak at 2θ = 42.9°. Fourier-transform spectroscopy of nanoB’s surface indicated the presence of C=O, C≡N–R, and C=C bonds, and other functional groups. The electron microscopic micrographs of nanoB showed cubical, pentagonal, needle, and spherical shapes. NanoB and nanoCu were applied alone and as a mixture at the rate of 1000 mg kg^−1^ soil, to pots where wheat crop was grown. NanoCu did not influence any soil or plant parameters except soil Cu content and plant Cu uptake. The soil and wheat Cu content in the nanoCu treatment were 146 and 91% higher, respectively, than in the control. NanoB increased microbial biomass N, mineral N, and plant available P by 57, 28, and 64%, respectively, compared to the control. The mixture of nanoB and nanoCu further increased these parameters, by 61, 18, and 38%, compared to nanoB or nanoCu alone. Consequently, wheat biological, grain yields, and N uptake were 35, 62 and 80% higher in the nanoB+nanoCu treatment compared to the control. NanoB further increased wheat Cu uptake by 37% in the nanoB+nanoCu treatment compared to the nanoCu alone. Hence, nanoB alone, or in a mixture with nanoCu, enhanced soil microbial activity, nutrient content, and wheat production. NanoB also increased wheat Cu uptake when mixed with nanoCu, a micronutrient essential for seed and chlorophyll production. Therefore, a mixture of nanobiochar and nanoCu would be recommended to farmers for improving their clayey loam soil quality and increasing Cu uptake and crop productivity in such agroecosystems.

## 1. Introduction

Nanotechnology is considered among the key emerging technologies that could pave the way to achieving sustainability in the agriculture sector [1]. This technology has developed nanostructured materials, such as nanopesticides, nanosensors, and nanofertilizers, with desired characteristics to achieve environmental friendly food production [2,3]. In the fertilizer sector, such improved properties control the nutrient release, deliver the nutrient to targeted sites, and thereby overcome the negative impact of conventional fertilizers [4]. Metallic nanomaterials or particles (MNPs), when utilized as fertilizers, their small size and customizable surface properties provide a platform for enhanced nutrient delivery [5]. The MNPs high surface to volume ratio enables them to provide several nutrient adsorption sites, that can retain nutrients in the soil and thereby reduce nutrient losses to the environment [6]. Therefore, nanofertilizers have the potential to maximize crop yields and reduce the negative impacts of agriculture, by overcoming environmental losses and decreasing fertilizer input [7].

The intensive use of MNPs in agriculture and other industries has released a large quantity of these materials into the environment. Currently, it is becoming evident that the uncontrolled release of MNPs causes serious threats to various forms of life in many ecosystems. These toxicological risks to soil organisms, and their accompanying below-ground functions, are mainly linked to the size, surface chemistry, and application rate of MNPs in the soil [8,9,10]. For instance, Navarro Pacheco et al. [11] tested different concentrations (1, 10, and 100 μg mL^−1^) of nanoCu on the immune cells of amoebocytes and earthworms in vitro. They found that the highest concentration (100 μg mL^−1^) of nanoCu decreased the sub-population of amoebocytes and earthworm coelomocytes. On the other hand, Zhao et al. [12] observed the influence of different doses (10, 100, 500 mg kg^−1^ soil) of nanoCu on the denitrification process, and activity and diversity of microorganisms. They observed that only 500 mg kg^−1^ of nanoCu reduced the denitrification rate and microorganisms responsible for this process, whereas other concentrations did not influence these parameters. Shi et al. [13] studied the influence of 10, 100, and 1000 mg kg^−1^ soil of nanoCu on paddy soil properties, and observed that concentrations of 100 and 1000 mg kg^−1^ nanoCu decreased the microbial biomass carbon, however, 10 mg kg^−1^ did not influence this parameter. So, it is evident from the literature that, a concentration of nanoCu higher than 100 mg kg^−1^ soil is toxic to soil organisms and their associated functions.

Many plant parameters were also affected by the application of nanoCu in the soil. Singh and Kumar [14] observed that both concentrations (100 and 1000 mg kg^−1^ soil) of nanoCu reduced fresh weight, root, and shoot length of *Raphanus sativus* L. seedlings. Similarly, the nanoCu concentration of ≥300 mg kg^−1^ soil treated rice did not produce grains, other concentrations (75 and 150 mg kg^−1^ soil) of nanoCu also did not increase Cu content in the rice grain, but decreased rice yield compared to a control [15]. Additionally, the application of 300 mg kg^−1^ nanoCu decreased soybean height, and protein content in the seeds [16]. These toxicity effects of nanoCu on soil organisms or plant cells are mainly linked to the physical contact of nanomaterials with the cell surface, metallic ion release, generation of reactive oxygen species (ROS) in the cell, and metabolic disorders [10,17]. NanoCu penetration into the cell membrane damaged the cell, and ROS-generated oxidative stress led to cell death [17]. On the other hand, adding a citric acid coating on nanoCu reduced their toxicity to soybean plants [16]. Hence, the application of ≥100 mg kg^−1^ nanoCu had a negative influence on plant growth and yield, however, a low concentration, or coating nanoCu with organic material, can reduce its toxicity to soil organisms and plants [13,15,16]. Therefore, nanoCu has the potential to be used as a nanofertilizer, for improving the nutritional quality of crops and increasing crop productivity, if utilized smartly [15,16].

Nanobiochar (nanoB) is the nanomaterial produced from pyrolysis of organic matter and ball milling techniques [18]. This organic nanomaterial has the potential to reduce the toxic effects of MNPs on soil organisms and plants [19]. For example, nanoB decreased the production of different enzymes, such as malondialdehyde and superoxidase dismutase, in the rice leaf [20]. The low activity of both enzymes is an indication that a plant cell is under normal conditions, with no or very little oxidative stress caused by ROS generation [21]. This evidence indicates that nanoB has the potential to mitigate plant stress caused by MNPs [20]. The nanoB possesses the ability to adsorb soil nutrients [22], moreover, this nanomaterial can immobilize harmful pollutants after its application to soil [23,24]. For example, application of 1% (10 g kg^−1^) nanoB reduced nitrate losses from the soil by 59.8%, compared to a control [25]. Likewise, this concentration of nanoB increased the dry weight of *Brassica chinensis* L. by 30%, compared to a control [26]. Hence, nanoB efficiently regulated the mobility of essential nutrients for plant growth, as well as natural and anthropogenic stressors and contaminants in the soil–plant system [23,27]. A reduction in the phytotoxic effects of different heavy metals was observed by Liu et al. [26], when they applied nanoB to *Brassica chinensis* L. in heavy metal contaminated soil. Such positive features of nanoB could help to mitigate the toxicity of MNPs in agriculture [26]. However, some recent studies in aquatic ecosystems have found that a high concentration of nanoB could also be toxic to aquatic life [28,29]. According to Huang et al. [28], a high concentration of nanoB (600 mg L^−1^) application adsorbed the nutrients and decreased their accessibility to algae, which hindered algal growth [28], but this effect was not observed in Cd-contaminated soil, even at a high nanoB application rate, of 1% [26]. Moreover, in another recent study, 0.3% root-zone and 3% foliar application of nanoB increased chlorophyll a and b, P, K, and N contents, by 3–4 fold in the shoots of *Daucus carota* L., compared to a control [30]. Hence, nanoB, even at high concentrations showed a positive influence on soil properties and crop productivity. Therefore, nanoB may have a potential to mitigate the toxic effect when applied together with MNPs, however no study has thus far investigated such effects.

The aims of this study were, (i) to synthesize nanoB from goat manure and (ii) to investigate the effect of high concentrations (1000 mg kg^−1^) of nanoCu and nanoB on soil microbial biomass, N, P, and K contents, and wheat N and Cu uptake; and (iii) to test the synergistic effects of co-mixing of nanoCu and nanoB on soil microbial biomass, nutrient availability, and wheat N and Cu uptake. We hypothesized that, nanoCu would decrease the microbial biomass in the soil; the presence of nutrients (N, P, K, and other) in nanoB would increase nutrient content in the soil and wheat N uptake; and that co-application of nanoB with nanoCu would decrease the toxic effects of nanoCu on soil microbial biomass, nutrient availability, and N uptake by wheat. To test these hypotheses, nanoB was synthesized from goat manure, using pyrolysis and ball milling methods. The treatments, nanoCu, nanoB, and their mixture were applied at (1000 mg kg^−1^ soil), in pots containing wheat crop, in clay loamy soil.

## 2. Results

### 2.1. The Crystallographic Structure of Nanobiochar

**Figure 1:**
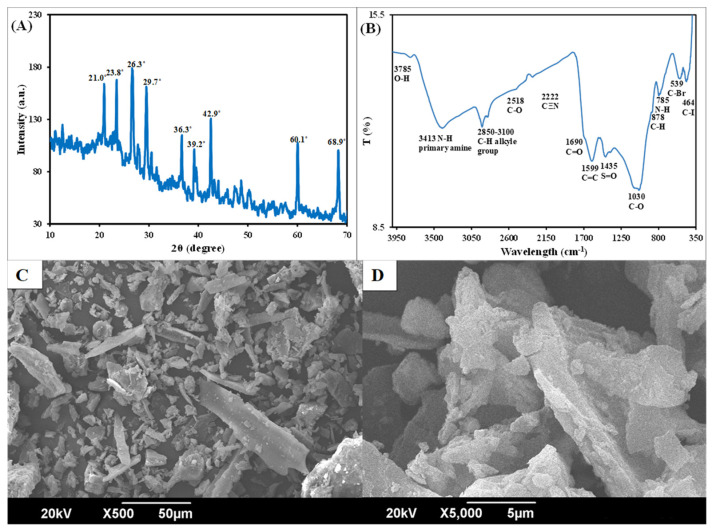
X-ray diffractogram (**A**), Fourier-transform infrared spectroscopy, T is the transmittance (**B**), and scanning electron microscopic micrographs at low- (**C**) and high-resolution (**D**), of nanobiochar.

The XRD spectrum showed strong and sharp peaks, indicating that the nanomaterial was well crystallized (Figure 1A). As the reaction continued, the peak intensities in the diffractogram became stronger. Such changes in the intensities might be linked to the diameter of particles, that are getting larger with increasing time. The observed peaks at various diffraction angles of the diffractogram showed the presence of different elements in the nanoB. The sharp peaks present between 20 and 30°, indicate the presence of carbon, and indexed at 002 of crystallographic plane. These sharp peaks show the high orientation of the crystallographic plane, which is established by parallel and azimuthal oriented –OH stretching of inorganic and/or organic partial carbonization of aromatic lamellae.

Another sharp peak, at 42.9°, also indicates the presence of graphene, a form of carbon, and indexed at 100, which is a condensed aromatic carbon crystallographic plane. Hence, these peaks indicate the presence of carbon in nanoB. The other small and sharp peaks at various 2θ angle locations indicate the presence of a number of minerals and elements in the nanoB. For instance, the sharp peak at 26.3° shows the presence of silica in the nanoB. The relatively small peaks at 39.2°and 45.1° show the existence of calcium compounds in the nanoB. The sharp peaks at 60.1° and 68.9° are probably due to the existence of hydrobiotite, kaolinite, and other minerals (Figure 1A). Hence, the XRD analysis indicated the presence of carbon and many other mineral compounds in the nanoB, such as calcium, magnesium, K, N, silicon, iron, sulfur, etc. The mean size of nanoB crystals, according to the Derby–Sherrer calculation, was 20.3 ± 3.4 nm.

The presence of different functional groups in nanoB was also observed through FTIR spectra (Figure 1B). This analysis showed the presence of aliphatic (C–H) and aromatic carbon (C=C) at 2850–3100 and 1599 cm^−1^, respectively (Figure 1B). Oxygen-containing functional groups including hydroxyl (–OH), at spectral peak of 3785 cm^−1^, carboxyl functional group (C=O), at the peak of 1690 cm^−1^, and sulfonyl (S=O) groups, at 1435 cm^−1^, were also detected in nanoB. A small peak observed at 2518 cm^−1^ showed the presence of an ester group (C–O–C), and the broad peak at 3413 cm^−1^ indicated the N–H stretching of primary aliphatic amines. A small peak observed at 2205 cm^−1^ indicated the nitrile group (C≡N–R) weak stretching vibration. The amide group is represented by the presence of a small peak at 785 cm^−1^, and strong peaks at 464–539 cm^−1^ showed the presence of halides in nanoB. The presence of these various functional groups can play a vital role in the soil ecosystem and ultimately crop performance.

The surface morphology, observed through scanning electron microscopic (SEM) analysis, indicated multimodal and polygonal shapes of nanoB (Figure 1C,D). These shapes were cubic, pentagonal, rod, or needle and spherical in nature (Figure 1A). The particles were agglomerated, making it difficult to measure the exact size. The mean estimated size of the nanoB particles was <1 μm (Figure 1D).

### 2.2. Soil Chemical and Microbiological Characteristics

**Figure 2:**
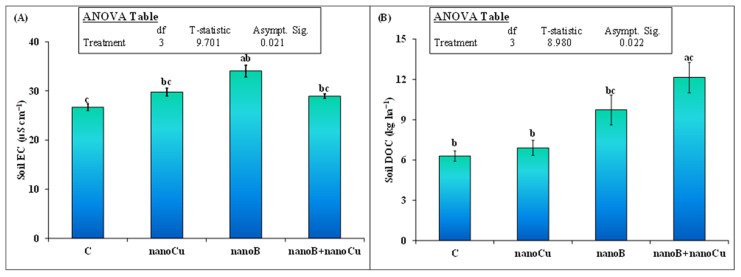
Mean (*n* = 3) (**A**) soil electrical conductivity (EC) and (**B**) dissolved organic carbon (DOC) at the end of the experiment, as affected by the sole and combined application of nanobiochar and nanoparticles. The control represents untreated soil. The inset table represents the outcomes of the nonparametric analysis of variance (ANOVA) after Kruskal–Wallis test. Error bars show standard errors (±1 SE) of the mean. Different small letters illustrate significant differences among treatments at a 5% probability level, after the Bonferroni correction for multiple tests.

**Figure 3:**
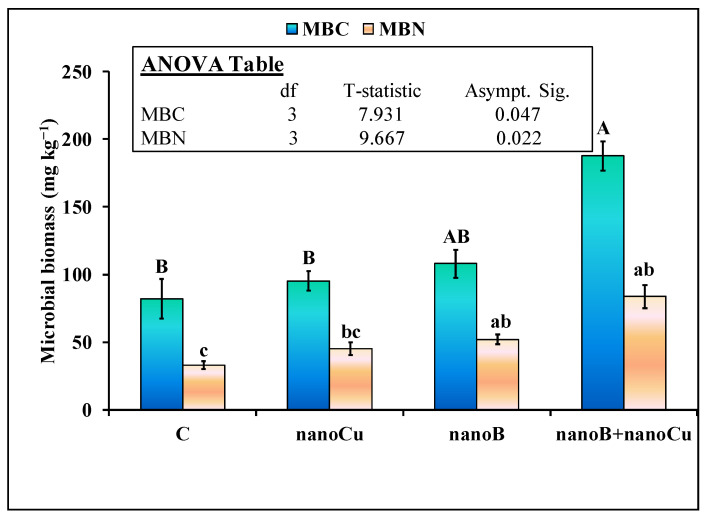
Mean (*n* = 3) microbial biomass carbon (MBC), and nitrogen (MBN), at the end of the experiment, as affected by the sole and combined application of nanobiochar and nanoparticles. The control represents untreated soil. Error bars show standard errors (±1) of the mean. The inset table represents the outcomes of the nonparametric analysis of variance (ANOVA) after Kruskal-Wallis test. Different capital or small letters illustrate significant differences among treatments at a 5% probability level after the Bonferroni correction for multiple tests.

Soil pH was not significantly affected by the treatment. None of the treatments (nanoB or nanoCu alone, or their combination) influenced this parameter in the soil at the end of experiment. The electrical conductivity (EC) of the soil was significantly increased by the application of both the nanoCu and nanoB treatments. The EC was 28% (34.0 vs. 26.7 µS cm^−1^) higher in nanoB than in the control. However, the nanoB and nanoCu mixture did not impact the EC in the soil (Figure 2A). Although dissolved organic carbon (DOC) was 54% higher in the nanoB treatment than in the control, it was not statistically significant. Interestingly, the nanoB and nanoCu mixture further enhanced the DOC content in the soil compared to nanoB or nanoCu alone, and the increment was 94% higher compared to the control (Figure 2B). NanoCu alone did not increase DOC compared to the control. Soil microbial biomass carbon (MBC) and nitrogen (MBN) were significantly affected by the studied treatments (Figure 3). The nanoCu and nanoB treatments did not influence MBC in the soil. However, the mixture of nanoCu and nanoB significantly increased the MBC in the soil, making it 129% higher than in the control, and 97 and 74% higher than for the nanoCu and nanoB treatments, respectively (Figure 3). NanoB alone increased MBN by 57% compared to the control. Moreover, the mixture of nanoB and nanoCu further increased MBN by 61% (84 vs. 52 mg kg^−1^) compared to nanoB alone. However, nanoCu alone did not increase MBN compared to the control (Figure 3).

**Figure 4:**
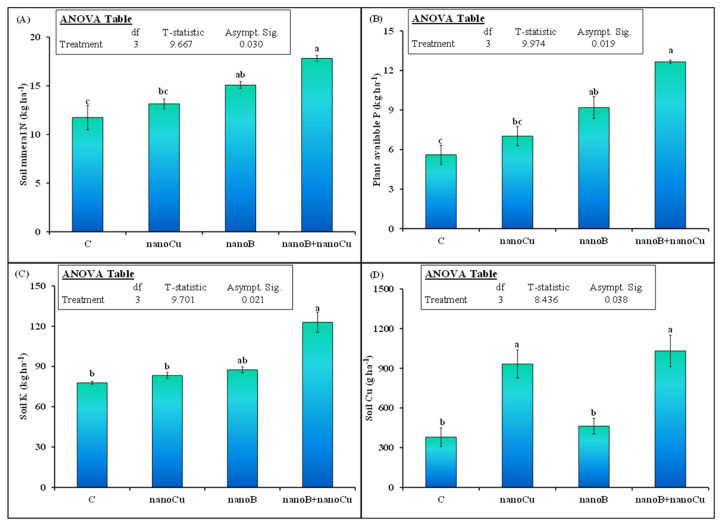
Mean (*n* = 3) (**A**) soil mineral nitrogen, (**B**) plant available phosphorus (P), (**C**) potassium (K), and (**D**) soil copper (Cu), at the end of the experiment, as affected by the sole and combined application of nanobiochar and nanoparticles. The control represents untreated soil. The inset table represents the outcomes of the nonparametric analysis of variance (ANOVA) after Kruskal-Wallis test. Error bars show standard errors (±1 SE) of the mean. Different small letters illustrate significant differences among treatments at a 5% probability level, after Bonferroni correction for multiple tests.

The soil macronutrient (N, P, and K) and Cu contents were also significantly influenced by the applied treatments (Figure 4). Interestingly, application of nanoCu did not influence any of the studied macronutrients (N, P, or K) in the soil, as compared to the control treatment, indicating that nanoCu was not toxic to nutrient mineralization in the soil (Figure 4A–C). On the other hand, nanoB increased N, P, and K in the soil by 28, 64, and 13%, respectively, compared to the control. Remarkably, in the mixture of nanoCu and nanoB treatment, the N, P, and K contents were 18, 41, and 38% higher, respectively, than for nanoB alone. These results indicate that application of nanoB synergistically increased the nutrient content and masked the toxicity (if that was caused by nanoCu) (Figure 4). Soil Cu content was 146% higher in the nanoCu alone treatment, compared to the control. The mixture of nanoCu and nanoB treatment further increased this parameter, by 123%, compared to nanoB alone (Figure 4D). However, nanoB did not alter the soil Cu content, showing that an increase in Cu was only observed where Cu was applied to the soil in the form of a nanomaterial.

### 2.3. Wheat Growth and Nutrient Uptake

Application of nanoCu or nanoB did not significantly affect the chlorophyll content in the wheat leaves of treated plants, compared to the control. In the mixture (nanoCu and nanoB) treatment, the chlorophyll content was 29% higher than in the control (Table 1). Plant heights were 11 and 25% higher in the nanoB and nanoCu+nanoB treatments, respectively, compared to the control. Similarly, spikelet number per spike, and biological yield of the wheat crop, were also affected by the nanoB alone and the mixture of nanoB+nanoCu treatments (Table 1). NanoCu alone did not influence these parameters of the wheat crop. Application of nanoB alone increased spikelet number per spike by 17%, whereas the mixture of nanoB and nanoCu increased this parameter by 33% compared to the control (Table 1).

Likewise, the biological (total crop dry matter) yield of the wheat crop was 25% higher in the nanoB alone treatment as compared to the control. Similarly, a mixture of nanoB+nanoCu increased this parameter by 35% as compared to the control. Spike length, root biomass, and harvest index were not affected by any treatment. NanoB or nanoCu alone did not increase number of grains per spike, 100 grains weight, or the grain yield significantly, compared to the control. Only the mixture treatment increased these wheat yield parameters compared to the control, and the increases were 28% (36 vs. 33 grains), 28% (5.6 vs. 4.4 g), and 62% (3483 vs. 2153 kg ha^−1^), respectively (Table 1). Hence, nanoCu alone did not influence most of the studied wheat parameters, however, nanoB alone, or in combination with nanoCu, improved the wheat productivity.

**Figure 5:**
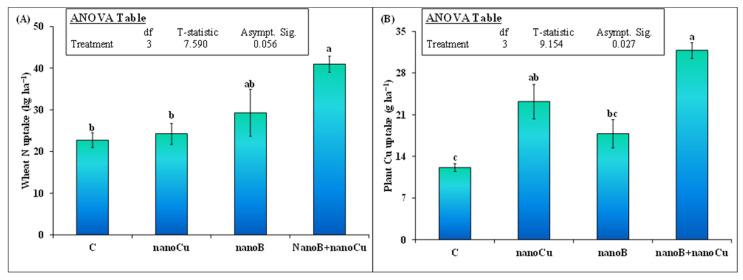
Mean (*n* = 3) wheat plant (**A**) N uptake and (**B**) Cu uptake after the sole and combined application of nanobiochar and nanoparticles. The control represents untreated soil. Error bars show standard errors (±1 SE) of the mean. The inset table represents the outcomes of the nonparametric analysis of variance (ANOVA) after Kruskal-Wallis test. Small letters illustrate significant differences among treatments at a 5% probability level, after Bonferroni correction for multiple tests.

The wheat N and Cu uptakes were also significantly influenced by all treatments (Figure 5). However, nanoCu and nanoB did not significantly increase wheat N uptake compared to the control. Remarkably, the mixture of nanoB and nanoCu increased wheat N uptake by 80% compared to the control. This was 40% higher than nanoB alone and 69% higher than nanoCu alone (Figure 5A). The wheat Cu uptake was only higher than the control in the treatment where nanoCu was applied (Figure 5B). In the nanoCu treatment, Cu uptake was 91% (23.2 vs. 12.1 g ha^−1^) higher than in the control. Similarly, the mixture of nanoB+nanoCu further increased wheat Cu uptake by 37% (31.8 vs. 23.2 g ha^−1^), compared to nanoCu alone. These results show that the mixture of nanoB and nanoCu is a promising fertilizer to improve the uptake of both macro- (i.e., N) and micronutrients (i.e., Cu) by wheat crops.

## 3. Discussion

We synthesized a nanobiochar (nanoB) to acquire unique characteristics such as small size, high surface area, charged surface, uniform pore distribution, and distinct morphology [31]. These properties are important in describing the extent and impact of nanoB in various applications [18,27]. Synthesis of nanoB in our study was confirmed by characterizing it using a number of techniques. The XRD and scanning electron microscopy analyses confirmed the crystalline nature of the nanoB, whereas the particle size of the synthesized material was in the nano range (Figure 1). Both XRD and Fourier-transform infrared (FTIR) spectroscopic analyses showed the presence of aromatic, organic, and inorganic compounds in the biochar surface. The FTIR analysis in our synthesized nanoB also confirmed the presence of oxygen, carboxyl, sulfonyl, and nitrile, etc., containing groups. These groups are important in removing a number of pollutants, through co-precipitation or complexation processes, from the soil or an aqueous medium [31], and adsorbing extra nutrients that are available in the soil after fertilization or organic matter decomposition [25,32,33]. For example, oxygen-containing groups on nanoB’s surface (Figure 1B) have been shown to provide suitable sites for NH_4_^+^ and NH_3_ adsorption [34], thereby increasing their availability in the soil for crop growth improvement [35]. Hence, nanobiochar addition in the soil would help to reduce nutrient losses and retain the nutrient in the soil, so that it might be available to the crop when required.

Many recent studies have shown that nanoCu, when applied at high concentration (≥ 100 mg kg^−1^ soil), was toxic to soil microorganisms, mesofauna, and macrofauna [11,12,13,36], as well as the soil biota associated processes of ammonification, nitrification, denitrification, and enzymatic activities [13,37]. Likewise, these high concentrations of nanoCu reduced seedling growth, plant height, grain yield, and ultimately crop production [14,15,16]. Therefore, we hypothesized that nanoCu application would decrease microbial biomass and the associated processes of nutrient availability in the soil. In contrast, we observed that nanoCu did not influence microbial biomass C and N in the soil compared to the control (Figure 3). In line with our study, Simonin et al. [38] also found that a high concentration of nanoCu did not influence microbial abundance and activity. They suggested that the low dissolution of nanoCu in agricultural soils might be the reason that no effect of this nanomaterial on microbial abundance was observed. Accordingly, Rippner et al. [39] found that the toxicity of nanoCu to soil microbial biomass was land-use dependent, and that the nanoCu did not cause toxicity to soil microbial biomass where agricultural practices were carried out. Instead, in their study nanoCu caused toxicity to microbial biomass in unmanaged soil [39]. Consequently, soil properties i.e., texture (sand, silt and clay content), organic matter, pH, cation/anion, and redox potential are important factors that determined the nanoparticles toxicity, fate and bioavailable nature in the soil [40]. For example, high dissolution of Cu from nanoCu caused more toxicity to bacterial communities in mineral than organic soil [41]. In addition, clay and organic matter content in the soil protected the microbial communities against the toxic effect of nanoparticles [42]. So, dissolution of the nanoCu and interference of soil properties with nanomaterials played a major role in determining MNPs toxicity [40]. In our study, Cu content in the soil was higher than control in nanoCu (Figure 4D) treatment indicating that dissolution of Cu may not play much role in causing toxicity to soil microbial biomass (Figure 2). Rather clayey nature of the soil might play a role and protect the microbes against toxicity of nanoCu as was observed by Pawlett et al. [42]. Therefore, in all probability, these might be the reasons, we did not observe toxicity of nanoCu to microbial biomass in our study.

In addition to microbial biomass, the nutrient availability (DOC, N, P and K content) in nanoCu amended soil was also not influenced (Figure 1 and Figure 4). This no increment in the nutrient availability in our study is in contradiction with Dimkpa et al. [43] who observed that MNPs increased the N and K level in the soil and sorghum plant. Similarly, in another study Raliya et al. [44] observed that ZnONPs increased the phytase and phosphatase enzymes that enhanced P solubilization and ultimately Mungbean P uptake. The relatively lower concentration of nanoCu 600 mg kg^−1^ enhanced essential nutrients in the soil for the growth of *Allium fistulosum* by improving antioxidant enzymes [45]. Interestingly, Simonin et al. [38] found that only 100 mg kg^−1^ nanoCu caused toxic effect to C and N cycling and explained that toxicity to microbes caused a decrement in N and C availability in the soil. Therefore, we can speculate that a high concentration of nanoCu did not influence nutrient availability in our study due to no impacts of NPs on microbes (Figure 3). Secondly, the clayey loam soil may adsorb the nanoCu on its surface and reduce nanoparticle access to microbes and its toxicity [42] to nutrient availability for plant uptake that are the most probable explanations, we did not observe an increase in N uptake in nanoCu amended soil than control (Figure 5A).

According to our expectation, we observed that nanoB alone increased microbial biomass N (Figure 3) and nutrient availability in the soil for plant uptake (Figure 4). Moreover, there was a tendency towards an increase in microbial biomass C and plant available K content in the soil, but these parameters were not statistically significant (Figure 3 and Figure 4D). The increment in microbial biomass could be linked to presence of high DOC and other mineral nutrients (mineral N and P) available in nanoB amended soil that might favor the microbial growth (Figure 2B, Figure 3 and Figure 4). Biochar and other similar fertilizers can serve as a food source of microorganism and thereby increase microbial biomass [46]. Accordingly, Liu et al. [26] observed that nanoB increased microbial biomass by providing favorable conditions for the growth of microbes. In another study it is observed that biochar based fertilizers increased nutrients such as N and DOC in the soil thereby increased microbial diversity and biomass [47]. In our study, the nanoB is produced from goat manure which is a rich source of the essential nutrients for crop growth (Table 2). When nutrient rich organic fertilizers were applied to the soil, they increased soil microbial activity, decomposition and nutrient mineralization of organic compounds present in the fertilizer and ultimately the nutrient availability for crops uptake in the soil [48,49,50]. So, mechanistically, in our study the nanoB provided rich source of nutrients as a food for microbes and increased microbial population [51]. Moreover, the interaction of nanoB with microbes can regulate the microbial associated processes of nutrient fixation [27], adsorption and mineralization/ mobilization of P and N in the soil [52]. Application of nanoB in the soil can adsorb mineral N and reduce its leaching and runoff losses thereby increased N retention in the soil [25]. These processes might occur in our study since we observed high DOC, mineral N, plant available P in nanoB amended soil than control (Figure 4). Such nutrient increment in this treatment led to increase plant height, spikelets per spike, and biological yield of wheat crop (Table 1) and also increased the wheat N uptake but the later parameter was not statistically significant (Figure 5).

Our third hypothesis, was that a mixture of nanoB with nanoCu would decrease the toxic effects of nanoCu on microbial biomass, nutrient availability in the soil, and wheat yield and N uptake. Interestingly, we did not observe a toxic effect of nanoCu on microbial biomass and other associated parameters, this might be due to the clayey nature of the soil used in our study. Remarkably, in the nanoB+nanoCu treatment, we observed much higher microbial biomass C, N, DOC, mineral N, and plant available P and K contents in the soil, as well as wheat biological, and grain yields and N and Cu uptakes, compared to nanoB or nanoCu alone treatments (Figure 1B, Figure 2, Figure 3, Figure 4 and Figure 5 and Table 1), indicating the synergistic effects of nanoB+nanoCu on these parameters. Such synergistic effects on soil microbial biomass could be explained by the better living conditions provided by the nanobiochar [26], especially under Cu deficient soil [53] like the one used in our study (Table 2). It is evident from some studies, that the right concentration of micronutrients can improve soil microbial growth, colonization, and N fixation [54,55]. Similarly, the addition of micronutrients in an N, P, and K fertilizer blend increased the soybean yield by 50%, compared to a normal fertilizer recommendation [56]. The co-application of different concentrations of Cu (0, 0.03, and 0.06 ppm) with P fertilizer significantly increased P utilization efficiency, compared to the application of P alone, showing the synergistic effect of Cu on P uptake in lettuce crop [57]. Accordingly, the application of nanoZn, nanoCu, and nanoB oxides stimulated soybean vegetative growth, as well as N and P accumulation in different vegetative parts of this plant [58]. These authors explained that crops usually demand essential nutrients at lower concentrations in the soil, including NPK and other micronutrients, to maximize their vegetative and/or reproductive development and growth [58].

The soil in our study was Cu deficient (Table 2), therefore, the mixture of nanoCu with nanobiochar provided suitable conditions for the microbial growth (Figure 3) and increased the macronutrients (N, P, and K) and micronutrient Cu (Figure 2B and Figure 4), since nanoB is a rich source of macro- and micronutrients [18]. This balanced nutrition of nanoB+nanoCu enhanced the vegetative growth of the wheat (Table 1), and their synergistic effects increased wheat N and Cu uptake, compared to the control, or nanoCu or nanoB alone, in our study (Figure 5). Our findings are also in accordance with Kihara et al. [53], who observed that maize Cu and N uptake was only increased when NPK and micronutrient Cu were applied in combination in Cu deficient soil. However, application of only Cu did not increase maize Cu uptake in their study, which is also in accordance with our results (Figure 5B), indicating that balanced nutrition is important for improving wheat productivity in micronutrient deficient soil.

The results obtained in our study are based on pot experiments with one soil and crop type, therefore, some restrictions of nanoB’s potential application to contaminated sites must be attached to it. For instant, texture (sand, silt, and clay), redox potential, organic matter content, pH, and cation/anion exchange capacity are the major factors responsible for the fate and bioavailable nature of nanomaterials in the soil [40]. So, nanoB’s potential reactivity to reduce toxicity of any contaminant in the soil mainly depends on these factors. However, the current study is performed in pots with one soil and crop type, therefore, its practical applicability in all soil and crop types may be limited. Therefore, a detailed mechanistic study, with the use of more soil and crop types, is suggested in the future, to confirm nanoB’s potential benefits in reducing the toxicity of novel contaminants in agroecosystems.

## 4. Materials and Methods

### 4.1. Nanomaterials

Commercial copper oxide nanoparticles were purchased in the form of powder from Sigma-Aldrich (St. Louis, MO, USA). The manufacturer provided the size and surface area of the nanoCu, which were < 50 nm and 23 m^2^ g^−1^, respectively. The same nanomaterial was also used in the study of Simonin et al. [38], who found that these MNPs had a mean size of 57 ± 18 nm and their zeta potential (surface charge) ranged between −13.8 and −21.3 mV, in different soil solutions.

### 4.2. Production of Nanobiochar from Goat Manure

The fresh goat manure was collected from a goat farm in the vicinity of Rawalpindi, Pakistan. After drying, in an open shady place, the manure was placed in a pyrolysis tank for 5 h at 500 °C [50]. The resultant biochar was extensively ground in a ball mill (F-P4000, Huanyu Instrument, Zhejiang, China) for 24 h at 300 rpm speed [59]. This machine consisted of a 500 mL volume tank and 800 stainless-steel balls, each with diameter of 3 mm, for grinding the material. Subsequently, the powdered biochar was sieved using a 200 µm mesh screen to obtain nanoB.

### 4.3. The Characterization of Nanobiochar

To confirm the nanoB synthesis, the material was characterized using different techniques to determine its surface morphology, surface properties, crystalline nature, and size. The morphology of nanoB’s surface was analyzed by scanning electron microscopy (SEM: S-4700, Hitachi, Japan). A sputtering technique was used to sputter gold particles onto the nanoB surface with sputtering apparatus (JEOL JFC-1500, Kyoto, Japan). The nanoB surface morphology was analyzed at a magnification range of 25–50,000× and 20 Kv.

The crystallinity of the nanoB was determined by scanning it with an X-ray diffractometer (STOE, Darmstadt, Germany). The radiation Cu-Kα-1 (λ = 0.15406 nm) was used for this purpose. The scanning angle 2θ was in the range of 10–70°, used at 0.4° step size per second. The Debye–Scherrer Formula (1) was used for calculating the crystal size of the nanoB.

(1) $$Size\;\left(nm\right)=\frac{K\lambda }{\beta \cos\theta }\;$$

where K is Scherrer’s constant, with a value of 0.94, the X-ray wavelength, λ, is 0.1546 nm, β denotes half diffraction (1/2) line’s radians, and θ denotes the half diffraction line angle.

A Fourier-transform infrared spectrometer (Perkin Elmer 100, MA, USA) was used to analyze the bonding nature of organic compounds present in the nanoB. Pellets of a nanoB and KBr mixture, as well as KBr alone, were made for the FTIR spectra analysis. The KBr pellet was used for correcting the background value. The nanoB was examined at wavelengths ranging from 400 to 4000 cm^−1^.

### 4.4. Pot Experiment

We carried out a pot experiment at an open space assigned for experiments in the Department of Agronomy, Pir Mehar Ali Shah, Arid agriculture university (PMAS-AAUR), Rawalpindi, Pakistan. The soil was collected from the university research farm. This soil was categorized as clayey loam, belonging to Rawal series, Udic Haplustalf Alfisols, according to the classification of soil survey of Pakistan. This soil was sieved, using a mesh size of 2 mm, to eliminate the damaged or broken parts of roots and stones. From this soil, we weighed 13 kg and filled a pot with it (area = 0.043352 m^2^). The diameter and height of the pot were 23.5 and 30 cm, respectively. The initial soil and nanoB properties are presented in Table 2. To fulfill the objective, the following four treatments were used (i) control (no fertilization), (ii) nanoCu at 1000 mg kg^−1^ soil, (iii) nanoB applied at 1000 mg kg^−1^ soil, (iv) a mixture of nanoB and nanoCu. The treatments were applied in triplicate. Wheat seeds were purchased from the local market and seventeen seeds were sown in each pot. The experiment was carried out in the last week of November 2020. After sowing, all treatments in pots were completely randomized and places in an open space, to provide a natural environment for the plants. The plants were regularly irrigated with a hand sprinkler, to avoid water shortage/stress, and the moisture level was maintained at 60% in each pot, by monitoring this with a low-cost soil moisture meter (FY -901, Hangzhou, China). The mean temperature and precipitation during the experimental period were 20.8 °C and 34.8 mm, respectively.

### 4.5. Chemical Analysis of Soil and Nanobiochar

In each pot, a hand auger was used to sample soil from the top 15 cm soil layer at three random spots, before sowing and after harvesting of the crop. These samples from each pot were thoroughly mixed. The composite sample was used for further biochemical analyses. For determining soil pH and electrical conductivity, we prepared a soil–water (1:2.5) suspension, after shaking the mixture on a linear shaker for 1 h. This suspension was left alone for 30 min to homogenize. A multi-meter (Multi 9430 IDS, WTW GmbH & Co., Oberbayern, Germany) was used for determining the pH and EC of this suspension. Soil mineral N was extracted with 2M KCl and then analyzed colorimetrically [33]. The plant available phosphorous (P) and potassium (K) contents were analyzed following the method of Houba et al. [60]. The total carbon content in nanoB and soil were determined by a wet oxidation method [61]. For this purpose, 1 g soil was weighed in a 250 mL volumetric flask, and then 10 mL K_2_Cr_2_O_7_ (1N) was added. After soil dispersion, 20 mL concentrated H_2_SO_4_ was added gently, to digest the soil sample. After digestion, the extract was titrated against FeSO_4_ in the presence of Ferroin indicator. A 5 g soil sample was weighed from the composite sample, then put in a 50 mL polytube to measure dissolved organic carbon (DOC). A volume of 25 mL distilled water was added to the polytube to make a suspension. These polytubes were placed in an incubator at 80 °C for the extraction of DOC. After filtering this suspension with a carbon-free filter, DOC was analyzed using a TOC analyzer (TOC-VCPH, Shimadzu, Kyoto, Japan) [62].

### 4.6. Microbial Biomass

A 10 g fresh soil sample was weighed, and divided into two equal halves for determining the microbial biomass C (MBC) and N (MBN). One portion of this soil was fumigated using 25 mL chloroform (CHCl_3_: ethanol free), in petri plates that were placed at the bottom of a vacuum desiccator, for CHCl_3_ fumes to kill microbes, at room temperature for 36 h. Then, these samples were placed in a water bath at 80 °C for 2h, to remove fumes. The fumigated and normal soil were put in 50 mL glass bottles for mixing with 25 mL K_2_SO_4_ solution (0.5 M), and mixed for half an hour on a reciprocal shaker at 250 rpm speed. A carbon-free filter was used to filter the resultant solutions. After this, the filtrate was examined for C content using a TOC apparatus (TOC-VCPH, Shimadzu, Kyoto, Japan), and total N through the Kjeldahl digestion procedure. Equation (2) was used for determining MBC and MBN:

(2) $$\mathrm{MBC}\;\mathrm{or}\;\mathrm{MBN}=\frac{C_{\mathrm{fum}}{\mathrm{or}\;N}_{\mathrm{fum}}-{\;C}_{\mathrm{nfum}}{\mathrm{or}\;N}_{\mathrm{nfum}}}{{\mathrm{kEC}}_{\;}\;\mathrm{or}\;\mathrm{kEN}}\;$$

where C_fum_, C_nfum_, N_fum_, and N_nfum_ show total C and N contents in fumigated and fresh soils, respectively. The kEC (0.45) [63] and kEN (0.54) [64] are the coefficients for MBC and MBN calculation, respectively.

### 4.7. Wheat Growth Attributes

At maturity stage (middle of April 2021), the wheat crop was cut at stubble height with a hand sickle. The fresh yield was determined by weighing the whole plants in each pot. The harvested material was oven dried at 70 °C, for two days, until a constant weight was achieved. Other physiological parameters were determined from three plants that were randomly selected from each pot. A Spad meter was used to determine the chlorophyll content from 10 independent observations on different wheat plants in each pot. The length of the panicle and height of the wheat plant were measured using a meter rod (cm). After counting the spikelets of selected plants, these were manually crushed, and the number of grains per spikelet/panicle were also counted. All plants from each pot were threshed manually, and the weight of 100 grains and total number of grains were measured, to obtain the grain yield of each pot. To determine the root biomass, a soil clump was taken out from the pot and placed in cold water for 4–5 h. Then, each clump was taken out and divided into four portions on a mesh sieve (1 mm). From each portion, we separated the roots from the soil using a tap water jet. These roots were placed in an oven for drying at 70 °C until a constant weight was reached. Then, the dry biomass was measured using a digital balance.

### 4.8. Wheat Nitrogen and Copper Uptakes

The crop dried shoot, root, and grains were ground together and then passed through a 1 mm sieve. The ground material was subjected to Kjeldahl digestion, and the N content was determined from the digested plant materials. For the Cu measurement, the ground plant material was digested with HNO_3_ and HClO_4_ (4:1) at 210 °C. Then the extract was subjected to atomic absorption spectroscopy (AA-7000, Shimadzu, Kyoto, Japan).

Nitrogen and Cu uptakes by wheat crops after treatment application, were calculated using the following equations:

(3) $$\mathrm{Nitrogen}\;\mathrm{and}\;\mathrm{Cu}\;\mathrm{uptake}\;\mathrm{unfertilized}\;\mathrm{treatment}=\left(N_{C0}\;\mathrm{or}\;{\mathrm{Cu}}_{C0}\times {\;\mathrm{DM}}_{C0}\right)$$

(4) $$\mathrm{Nitrogen}\;\mathrm{and}\;\mathrm{Cu}\;\mathrm{uptakes}\;\mathrm{in}\;\mathrm{nanomaterial}\;\mathrm{treatments}\;=\left({\;N}_{f}{\;\mathrm{or}\;\mathrm{Cu}}_{f}\times {\;\mathrm{DM}}_{f}\right)\;$$

where N_f_ and Cu_f_ designate total N and Cu contents in the wheat shoot or root of nanomaterial treatments (kg N and Cu). DM_f_ is the dry matter yield (kg ha^−1^) of wheat in nanomaterial treatments. N_C0_ and Cu_C0_ indicate N and Cu contents observed in the control treatment. DM_C0_ is the wheat dry matter yield in control (kg ha^−1^).

### 4.9. Statistical Analysis

The influence of the treatments on the soil nutrient content and EC, as well as wheat crop dry matter yield, N, and Cu uptake were subjected to statistical tests using the statistical package for social studies (SPSS 20, IBM, Armonk, NY, USA). The normality of the data was analyzed by Kolmogorov–Smirnov and Shapiro–Wilk tests. Since the data was not normal, a nonparametric analysis of variance (ANOVA), using independent samples, Kruskal–Wallis test was used to study the influence of different treatments on the above parameters, at 5% probability level. Where necessary, multiple comparisons among treatments were made using Bonferroni correction for multiple tests.

## 5. Conclusions

This is the first study comparing the influence of relatively higher concentrations of nanoCu and nanoB (1000 mg kg^−1^ soil) alone, and as a mixture, on microbial biomass, mineral N, P, and K contents in the soil, and wheat N and Cu uptake. Our results indicated that nanoCu or nanoB treatments alone did not cause toxicity to these studied parameters. Interestingly, nanoCu alone did not influence any of the soil or plant studied parameters. NanoB increased microbial biomass, mineral N, and plant available P in the soil, as well as wheat N uptake, which could be ascribed to the nutrient-rich nature of the organic parent material used to synthesize it. The nutrient-rich nature of nanoB was responsible for improving the soil quality and wheat productivity. Moreover, the mixture of nanoB+nanoCu synergistically influenced microbial biomass and crop available micronutrients (N, P, and K) in the soil, compared to application of either nanoB or nanoCu alone, indicating that a balanced nutrition, consisting of macro- and micronutrients, is complementary for achieving good soil quality and wheat productivity in micronutrient deficient soil, which was the case in our study. Therefore, the co-mixture of nanobiochar and CuO nanoparticles (nanofertilizer) would be recommended for farmers to improve the soil quality and crop productivity, especially in clayey soil under micronutrient deficiency. This is a short term study, with the use of only one soil and crop type, therefore long-term field studies (more than one growing seasons) are required, with more soil and crop types, to confirm the reliability of our results.

## Figures and Tables

**Table 1:** Mean (*n* = 3) physiological and yield attributes of the wheat crop, as influenced by the control (unfertilized), nanobiochar (nanoB), and CuO nanoparticles (nanoCu). Harvest index is the ratio of wheat grain yield to biological yield (total crop dry matter). Abbreviations of fertilizer treatments can be seen in Figure 1. Different small letters in a row illustrate significant differences among treatments at a 5% probability level after the Bonferroni correction for multiple tests.

Parameters	Units	C	nanoCu	nanoB	nanoCu + nanoB
Chlorophyll content	SPAD	32.0 ± 1.2 ^b^	34.0 ± 3.1 ^ab^	38.4 ± 2.7 ^ab^	41.4 ± 1.8 ^a^
Plant height	cm	57.6 ± 1.8 ^c^	60.7 ± 1.7 ^c^	63.9 ± 0.4 ^bc^	71.9 ± 1.7 ^a^
Spike length	cm	8.1 ± 0.6	8.2 ± 0.1	8.7 ± 0.8	9.3 ± 0.6
Spikelet	Number spike^−1^	15.2 ± 0.9 ^c^	16.5 ± 0.8 ^bc^	17.8 ± 0.3 ^abc^	20.3 ± 0.9 ^a^
Grains	Number spike^−1^	25.6 ± 1.0 ^b^	26.2 ± 2.8 ^ab^	29.2 ± 1.9 ^ab^	32.7 ± 1.5 ^a^
100 grains weight	g	4.4 ± 0.4	4.5 ± 0.2	4.9 ± 0.1	5.6 ± 0.3
Root biomass	kg ha^−1^	446.0 ± 42.8	469.0 ± 55.4	522.9 ± 111.7	676.6 ± 94.5
Biological yield	kg ha^−1^	5282 ± 400 ^c^	5636 ± 412 ^bc^	6616 ± 462 ^ab^	7135 ± 303 ^a^
Grain yield	kg ha^−1^	2153 ± 131 ^b^	2476 ± 169 ^ab^	3076 ± 431 ^a^	3483 ± 474 ^a^
Harvest index	%	40.9 ± 0.7	44.3 ± 3.6	46.7 ± 6.0	49.6 ± 8.9

**Table 2:** Mean (*n* = 3) of initial chemical properties (i.e., pH, electrical conductivity (EC), mineral N, total N, P, K, plant available P (PAP), plant available (PAK), Cu content, microbial biomass C (MBC) and N (MBN) of soil and nanobiochar (nanoB). - shows data of the selected parameter was not determined.

Parameters	Units	Soil	nanoB
pH		7.70 ± 0.07	6.74 ± 0.18
EC	uS cm^−1^	24.47 ± 0.38	-
Total organic C	g kg^−1^	0.71 ± 0.10	44.5 ± 2.294
Dissolved organic C	mg kg^−1^	1.73 ± 0.11	-
Mineral N	mg kg^−1^	3.73 ± 0.37	769.04 ± 66.13
Total N	%	-	5.80 ± 0.17
Total P	%	-	3.75 ± 0.15
Total K	%	-	3.11 ± 0.20
PAP	mg kg^−1^	1.13 ± 0.13	-
PAK	mg kg^−1^	30.60 ± 0.78	-
Cu	mg kg^−1^	0.12 ± 0.03	0.16 ± 0.02
MBC	mg kg^−1^	61.70 ± 8.87	-
MBN	mg kg^−1^	24.99 ± 1.87	-

## Data Availability

All the data is presented in the manuscript.
